# Moral Judgment: An Overlooked Deficient Domain in Multiple Sclerosis?

**DOI:** 10.3390/bs8110105

**Published:** 2018-11-16

**Authors:** Samar S. Ayache, Moussa A. Chalah

**Affiliations:** 1 EA 4391 Excitabilité Nerveuse et Thérapeutique, Université Paris-Est, 94010 Créteil, France; samarayache@gmail.com or samar.ayache@u-pec.fr; 2 Service de Physiologie-Explorations Fonctionnelles, Hôpital Henri Mondor, Assistance Publique—Hôpitaux de Paris, 94010 Créteil, France; 3 Neurology Division, Lebanese American University Medical Center-Rizk Hospital (LAUMC-RH), 1100 Beirut, Lebanon

**Keywords:** multiple sclerosis, moral decision-making, social cognition, alexithymia, theory of mind, empathy

## Abstract

Multiple sclerosis (MS) is a chronic inflammatory and neurodegenerative disease of the central nervous system through which patients can suffer from sensory, motor, cerebellar, emotional, and cognitive symptoms. Although cognitive and behavioral dysfunctions are frequently encountered in MS patients, they have previously received little attention. Among the most frequently impaired cognitive domains are attention, information processing speed, and working memory, which have been extensively addressed in this population. However, less emphasis has been placed on other domains like moral judgment. The latter is a complex cognitive sphere that implies the individuals’ ability to judge others’ actions and relies on numerous affective and cognitive processes. Moral cognition is crucial for healthy and adequate interpersonal relationships, and its alteration might have drastic impacts on patients’ quality of life. This work aims to analyze the studies that have addressed moral cognition in MS. Only three works have previously addressed moral judgement in this clinical population compared to healthy controls, and none included neuroimaging or physiological measures. Although scarce, the available data suggest a complex pattern of moral judgments that deviate from normal response. This finding was accompanied by socio-emotional and cognitive deficits. Only preliminary data are available on moral cognition in MS, and its neurobiological foundations are still needing to be explored. Future studies would benefit from combining moral cognitive measures with comprehensive neuropsychological batteries and neuroimaging/neurophysiological modalities (e.g., functional magnetic resonance imaging, tractography, evoked potentials, electroencephalography) aiming to decipher the neural underpinning of moral judgement deficits and subsequently conceive potential interventions in MS patients.

## 1. Introduction

Multiple sclerosis (MS) is a chronic progressive disease of the central nervous system (CNS) characterized by demyelination, synaptopathy, and neurodegeneration involving the brain and spinal cord [1,2,3]. It is the main cause of nontraumatic disability in young adults [1]. Its precise etiology remains unclear and includes a constellation of mechanisms. The mobilization of peripheral immune cells and their access to the CNS through an impaired blood-brain barrier appears to play a key role in MS pathogenesis, based on studies showing mainly macrophages and CD8+ T cells but also CD4+ T cells, B cells, and plasma cells in MS lesions [2,3,4].

MS can clinically manifest as three types: the relapsing remitting (RR) type, which is characterized by periods of acute symptoms onset separated in time by periods of full or partial recovery; the primary progressive (PP) type, which entails a steady progressive evolution of the disease since its onset; and the secondary progressive (SP) type, which is the conversion of the RR type into a pattern of progressive clinical worsening [2,4,5]. During the course of the disease, patients can report motor, sensory, and cerebellar symptoms but can also suffer from cognitive, emotional, and behavioral manifestations [1,2,6,7,8,9,10,11,12,13].

Although cognitive deficits are common in MS patients, affecting up to 65% of them, little attention has been paid to cognitive and behavioral performance in this population [13]. Among the most frequently impaired cognitive domains are attention, information processing speed, and working memory, which have been extensively addressed in this population. However, less emphasis has been placed on social cognition, which entails the individual’s abilities to recognize others’ emotions, intentions, and beliefs (i.e., facial and bodily emotion recognition, theory of mind (ToM)) and the capacity to empathize with others (i.e., empathy). Social cognition is crucial for healthy social functioning and deficits in this capacity may affect the quality of life [13]. Social cognitive deficits might be behind altered social interactions, high prevalence of social anxiety, and increased rates of unemployment and divorce observed among MS patients [13]. Moreover, some of the latter issues could result from a social reconfiguration within a family, where healthy members would face the obligation to deal with MS-related physical and cognitive consequences [14].

In addition to social cognition deficits, MS patients also exhibit high prevalence of alexithymia, which is a multicomponent personality trait implying difficulties to understand and describe one’s emotions and an externally oriented thinking (EOT) [8]. Although this trait can affect around 10% of the general population, its prevalence in MS patients can reach 53% [8]. This highlights the difficulties of MS patients to understand their own emotions as well as to understand others’ emotions and to subsequently empathize with them [8].

Besides these socio-affective domains, moral judgment is a complex cognitive sphere that enables individuals to judge others’ actions. It is defined by the set of habits and values that orient the social conduct in a certain group [15]. Moral reasoning relies on conscious processes in charge of transforming given information about actions by comparing them to a set of virtues, with the aim of attaining a moral judgement [15]. If deficits in moral judgment occur in MS, they would be very debilitating for patients and their social circle.

The main objective of the present work is to shed light on studies that assessed moral judgment in patients with MS. We will first define the selection criteria of this work. Afterwards, a brief overview of the available data on the underlying mechanisms of moral cognition and its assessment will be presented. This will be followed by an analysis of the studies that considered moral judgment in MS. Plausible underlying mechanisms of moral judgment deficits in MS will be tackled in the light of the available findings. Finally, some recommendations will be provided for future studies in order to improve the current understanding of these deficits.

## 2. Study Selection

**Figure 1:**
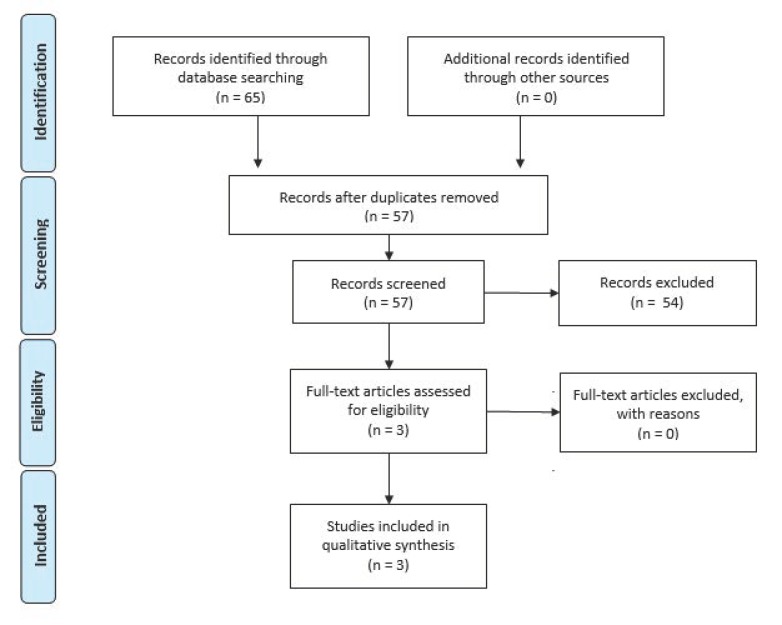
Flow diagram of the adapted research method.

Computerized databases (MEDLINE/PubMed, Scopus) were consulted, and a search was conducted independently by both coauthors according to the Preferred Reporting Items for Systematic Reviews and Meta-analyses (PRISMA) guidelines [16] with the aim to identify original research articles published at any time until 28 August 2018, in English and French languages, regarding moral judgement in MS patients. The following key terms were used: (‘moral judgement’ OR ‘moral judgment’ OR ‘moral cognition’ OR ‘moral competence’) AND (‘multiple sclerosis’ OR ‘MS’). Both authors screened the titles, abstracts, and full texts of all references retrieved in the searches and determined the eligibility and possible inclusion of each article. In cases of uncertainty, the full text of the manuscript in question was assessed by both authors and a final decision was made concerning inclusion/exclusion. Additional citations were searched by scanning the references of selected papers. Three publications matched the selection criteria and consisted of case-control studies that compared moral judgment between MS patients and healthy controls. They also included some neuropsychological measures, but none assessed the neurobiological underpinnings of moral judgment by means of neuroimaging or neurophysiological modalities. A flow diagram of the research method is illustrated in Figure 1 [16].

## 3. A Brief Overview of the Neurobiology of the Moral Brain

Processing morality seems to involve complex brain networks that exhibit a certain degree of overlapping with those devoted to other emotional, cognitive, and behavioral processes, including basic (e.g., attentional control, executive functions) and social cognition (i.e., emotion processing, ToM, empathy) [17]. These circuitries involve cortical (e.g., frontal, temporal, parietal, and cingular regions) and subcortical (e.g., basal ganglia and amygdalo-hippocampal complex) regions [17,18]. In MS, no structural or functional imaging studies have assessed the neurobiological correlates of moral judgment. For this reason, this section will briefly reappraise the cerebral substrates of this domain as derived from studies in healthy subjects and clinical populations other than MS.

Nowadays, one of the most accepted models in philosophy, psychology, and biology research explains moral cognition in the light of the dual-process theory, which is supported by clinical and behavioral data [17,19,20]. The theory entails the existence of two distinct systems that compete during the generation of a moral judgement [17,19]. The first one is an automatic, quick, intuitive, and emotion-driven deontological system with feature-specific sensitivity during a situation; the second one is a slow, cognitive, explicit, and deliberative system that reasons about utilitarian consequences [19,21,22,23,24,25,26]. In other words, a second reasoning system is deployed to correct the initial intuitions or emotional impulses.

However, the mode of interaction between these two systems remains controversial [17]. In fact, both systems seem to have non-overlapping dissociable neural substrates that work in an independent and parallel manner. In other words, judging a moral dilemma seems to involve, on the one side, the orbital and ventromedial parts of prefrontal cortex (PFC) and, on the other side, areas such as the dorsolateral PFC and the parietal cortex [27]. While the former areas deal with the autonomic emotional aversion to harm, the latter influence the cognitive rational control process; a process that engenders a cognitive propensity and maximizes the welfare regardless of cost [17,18,21,27,28,29]. In this context, the anterior cingulate cortex (ACC) intervenes to ensure that the conflict between the ventromedial and dorsolateral PFC is solved [17,21,27].

Besides the abovementioned dual-process model, other authors, like Cohen and Ahn, propose a single-process model, which they called ‘the subjective utilitarian theory’ of moral judgment [20]. It entails the presence of a single process by which an individual makes moral decisions. Here, the competition will occur between similarly valued items rather than between two distinct decision processes (e.g., a moral dilemma denoting the death of one’s own child versus the death of five of his/her friends). The proposed system is concerned with identifying and saving the item with relatively higher personal value [20] (i.e., the child would likely survive in the proposed dilemma). The more the items resemble each other, the more difficult the decision is.

Furthermore, some researchers consider that moral cognition puts into action multiple processes, which may challenge the previous models and propose a shift toward a ‘dynamic system model’ of moral cognition [30]. One of the arguments supporting this notion lies in the fact that the dual-process theory cannot fully explain, for instance, why modulating/altering the function of the dorsolateral PFC yielded opposing results across the studies (i.e., resulting in more utilitarian judgment versus less utilitarian judgment depending on the studies) [31]. Such a finding suggests an ‘integration-and-selection’ function rather than a restricted ‘rational cognitive control’ function of the dorsolateral PFC [31]. In other words, instead of exclusively having a cognitive control function, the dorsolateral PFC may be able to select a specific moral response among the available options and integrate information about utilitarian consequences and moral rights with dilemma-specific contents following moral rules. Based on these findings, moral judgment may constitute a dynamic process that puts into action several mental computations that are devoted to processing self-related (e.g., personal goals and identities) and others-related information (e.g., others’ mental states, social norms, social categories, reputational information) [30]. In a recent activation likelihood estimation metanalysis, the authors concluded that moral judgment recruits (i) a series of brain areas (e.g., medial PFC, lateral orbitofrontal cortex, temporoparietal junction (TPJ), amygdala, precuneus) that are common to all moral tasks and (ii) unique networks devoted to each of the moral modalities such as mentalizing method (affective versus cognitive ToM), instructional cues (instructing the individual to focus on morally (explicit) versus non-morally (implicit) salient information), role (i.e., self versus other, victim versus agent), and proximity (i.e., psychological versus imagined physical distance separating perpetrator from victim) [31]. Other works also found that a higher engagement in ToM abilities (recruitment of TPJ, precuneus and dorsomedial PFC) occurs when facing psychological versus physical harm, although both types activate the same brain regions [32,33]. These findings support the concept of a dynamic and multi-process moral judgment network and might account for the difference in brain activation pattern across moral judgment studies.

Adding to studies that explored the neural substrates of moral cognition, few available physiological reports have proposed a link between autonomic physiological responses and morality. For instance, utilitarian moral judgment was found to be associated with low heart rate variability (low cardiac vagal tone) [34,35] or low ability to generate skin conduction response (a somatic index of affective state/autonomic arousal) [36,37]. This perhaps reflects low neurovisceral integration at the level of PFC at the basis of the utilitarian pattern of response. Conversely, deontological moral judgment was found to be associated with high peripheral vasoconstriction/total peripheral resistance, a vascular measure that may reflect good/high visceral reactions [38].

It is also worth noting that some variables might contribute to moral judgment such as genetic variations (e.g., CAG polymorphism in androgen receptor gene [39], oxytocin receptor gene [40,41]), neuroendocrine factors (e.g., neuropeptide oxytocin [42], hypothalamic–pituitary–adrenal (HPA) axis [18]), neurotransmitters (e.g., serotonin, dopamine, noradrenaline [18,43,44,45,46]), environmental conditions (e.g., geographical area, climate, stress), and sociocultural conditions (e.g., gender) [18,47,48].

## 4. Exploration of Moral Judgment

Quantifying moral judgment is possible via several tools such as moral dilemmas [36]. These dilemmas consisted of situations where every possible course of action would breach some otherwise binding moral principles [49]. Common adopted scenarios involve harming an individual for the welfare of a group of subjects. Accepting such a behavior may reflect a utilitarian pattern of moral judgment (utility of scarifying one person for the sake of sparing a majority), which is driven by a detailed cost-benefit analysis, while unaccepting such a behavior might derive from an instinctual aversion toward harm [21,27].

The ‘Footbridge’ and the ‘Trolley’ stand among the most famous and ubiquitous moral dilemmas [37,49,50,51,52]. Both well illustrate the conflict between the utilitarian and deontological appraisals. The ‘Footbridge’ dilemma proposes two responses: (i) a utilitarian one that is supported by cognitive processes and favors pushing someone off of a foot bridge into the path of a runaway trolley in order to save five lives and (ii) an alternative nonutilitarian response driven by automatic emotional processes and entails sparing the man and allowing the others to die [23,53,54]. In the ‘Trolley’ dilemma, individuals have to choose between (i) doing nothing and allowing the trolley to move forward and kill five people tied-up on its track (nonutilitarian response), or (ii) redirecting the track by hitting a switch, this action would save the attached people but would result in scarifying the life of a single person lying on the side track (utilitarian response). While the Footbridge dilemma is personal (directly killing the man by pushing him into the path of a trolley), the Trolley dilemma is impersonal, since the decision of the responder would indirectly result in killing an individual by pulling a lever that will stop an arriving carriage that would otherwise kill five people [27,29,55,56].

Other moral dilemmas propose some situations/stories where an agent cannot fulfill the moral requirements in question. Here, participants are usually asked to perform several ratings including their degree of acceptance of the agent’s behavior (i.e., moral acceptability/permissibility), the pleasantness/unpleasantness of the experience (i.e., emotional valence), and their response to the exposed scene (i.e., emotional arousal/reactivity) [57]. Besides these dilemmas, scrutinizing moral judgement is feasible by presenting pictures or visual sentences [21,58,59] or by employing questionnaires [60].

## 5. Moral Cognition in Multiple Sclerosis Studies

Three studies have addressed moral cognition in patients with MS [61,62,63]. In the first one [61], Gleichgerrcht and colleagues employed a series of moral vignettes adapted from Greene and colleagues’ battery of moral dilemmas [21,27]. The stories assess (i) the individual’s ability to accept hurting others in an attempt to benefit a majority of persons (moral permissibility), (ii) the amount of one’ emotional reaction in front of a moral dilemma (emotional reactivity), and (iii) the extent of the individual’s egocentric perception of others’ attitudes with regard to presented moral scenarios (i.e., to which extent others may rate the act in a similar manner: moral relativity). The study also included measures of alexithymia and empathy. Compared to healthy controls, MS patients exhibited altered moral judgment, high alexithymia, and low empathy scores. They significantly differed from healthy controls by having a decreased moral permissibility as well as increased moral relativity and emotional reactivity. Results on moral dilemmas among patients may be related to socio-emotional domains such as alexithymia and empathy, based on some evidence supporting a relationship between alexithymia, social cognition, and moral judgment [64,65,66,67]. However, it is important to note that the direction of this relationship is somehow intriguing in MS patients since, in other clinical populations, low empathic abilities and high alexithymia were linked to utilitarian rather than deontological moral judgment [29,68,69,70,71,72,73], warranting further explorations.

Another contributory factor to low moral permissibility might be stress, which was previously found to correlate with less utilitarian moral judgment [48]. In fact, due to the unpredictable course and events in MS, the latter constitutes a chronic stressful condition through which patients may frequently experience fears and anxiety related to the uncertainty of the disease consequences. For instance, they might be afraid of getting irreversible disability during an MS relapse and might be afraid to experience a new MS attack while they are in remission [62]. In this context, the activation of stress response might have dictated the appearance of non-utilitarian judgment in this cohort.

From a neurobiological perspective, the dual-process model may suggest that the non-utilitarian pattern reported in MS patients is due to higher ventromedial PFC and/or lower dorsolateral PFC activity, especially in considering that MS pathophysiology is known to affect the frontal networks [74,75,76]. However, it is difficult to confirm this speculation since no MS study has included neuroimaging measures to examine the link between frontal circuits integrity and moral judgment capacities. Nevertheless, some studies have addressed the neural substrates of social cognition, which may be also shared by and/or contribute to moral judgment performance. These works have linked social cognitive deficits to diffuse white matter pathologies [77,78,79,80] as well as gray matter atrophy (involving the amygdala [81], cingulate cortex [79], left temporal pole, and left fusiform facial area [80]). Additional evidence has been derived from functional magnetic resonance imaging (MRI) studies where patients’ performance on social cognition tasks was associated with abnormal pattern of activation in the left insula (hypoactivation [77]), left ventrolateral PFC (hypoactivation [77] or hyperactivation [82]), and precuneus and posterior cingulate cortex (hyperactivation [83]) or decreased functional connectivity between the amygdala and the ventrolateral/medial PFC [82]. Admitting the relationship between social and moral cognition, these brain areas might overlap with those devoted to moral cognition and merit future research. In addition, other factors may have also contributed to patients’ performance, including neuroendocrine dysfunction and neurotransmitters imbalance, since the latter have been previously described in MS [7] and were found in some studies to be associated with moral judgment performance [18,43,44,45,46].

Besides moral permissibility, heightened emotional reactivity might be related to a dysfunction of emotion regulation, which has been previously reported in MS [84,85,86,87]. It might also be related to arousal/autonomic reaction. Therefore, it could be interpreted in the light of the aforementioned neurophysiological studies that addressed moral cognition, which featured a relationship between high physiological reaction and non-utilitarian moral judgment [38]. This relationship could explain the observed findings (i.e., low permissibility) in this MS cohort.

A second study by the same team was interested in understanding MS patients’ attitudes towards third-party violations [62]. Patients were exposed to 24 unique stories adapted from Young and colleagues’ work [88]. After each scenario, the MS patients were asked to rate (i) how much the agent’s action was appropriate, (ii) how severe the agent should be punished, and (iii) to which extent other people may respond to this scenario in a similar manner as them. Similar to the previous work, patients reported higher alexithymia ratings and lower empathy scores compared to healthy controls. Although they did not differ from healthy controls in their moral judgment of some acts, they had higher levels of emotional reactivity, judged others’ behavior to be less appropriate, and attributed more punishments for them. Such an outcome was driven by the EOT dimension of alexithymia, which could be defined as a concrete, introspection-devoid, reality-based, and literal thinking along with a tendency to avoid active conflict resolution [8]. Again, some research has suggested that, in the face of the traumatic nature of the disease, one of the adapted coping strategies by MS patients could be to orient their thinking on external events rather than paying attention to their inner feelings [13,89,90]. Therefore, EOT may emerge as a cognitive adaptive strategy that protects individuals from MS-related stress by avoiding self-reflection, distracting attention from self-oriented ruminations, and blunting the influence of negative arousal states [91,92]. Another plausible explanation would be that stricter moral attitude toward others could be related to frequently encountered symptoms in MS such as fatigue, depression, anxiety, and deficits in ToM or other cognitive domains, all of which were not addressed in this work and deserve to be addressed in future works [6,7,8].

In a third recent study, Realmuto and colleagues reported that their MS patients had comparable moral judgment performance relative to healthy controls but exhibited lower moral permissibility on instrumental dilemmas [63]. However, unlike the previous works, which documented high emotional reactivity in MS patients, the third study reported low emotional arousal in the recruited cohort. Such a finding may reflect emotional detachment, which might serve as a coping strategy aimed toward adapting to social contexts and maintaining a certain quality of life [93]. Moreover, levels of empathy and alexithymia, both of which were associated with moral performance in the previous two studies, were not assessed in this third study and might have mediated the observed discrepancy in emotional response. The difference in employed moral stimuli across these studies may constitute an additional explanation for the observed mismatch in emotional arousal/reactivity.

A summary of these studies is available in Table 1.

## 6. Current Conclusions and Future Perspectives

Based on the very few available data, MS patients seem to have a different pattern of moral judgment compared to healthy individuals. More research is obviously needed to be able to replicate the observed pattern of moral judgment in large MS cohorts. Of note, the three examined cohorts consisted of patients suffering from RR MS and having low disability scores. The hallmark of the latter disease type is inflammation while PP and SP MS types are characterized by the predominance of neurodegeneration [7]. In fact, cognitive deficits are more pronounced in patients with progressive MS and the more severe levels of cognitive decline seems to happen during the progressive phase of the disease [106], prompting an investigation of morality in these MS phenotypes. Another issue concerns the disease modifying therapies, which may have some effects on cognitive functions [107] and deserve to be addressed as potential confounders in relation to moral cognition.

In addition, the cohorts consisted predominantly of adult women. Gender difference has been previously documented with regards to brain connectivity pattern, alexithymia, empathy, as well as moral judgment [8,108,109]. The impact of this factor is worth considering in future research, since gender and other sociodemographic variables, such as age and cultural differences, may lead to different patterns of moral judgment [48,110]. For instance, in one study, more utilitarian judgments were found among young participants compared to older ones [47], among men compared to women [47,48], and among Western men compared to Eastern men [47]. That is to say, differences across cultures may include regulatory social institutions (e.g., economic markets, kinship structures) and social ecology (e.g., population density, residential mobility, prevalence of pathogens, weather and other environmental factors) [110]. Genetic variation may also contribute to study outcomes [17,18].

Therefore, future studies could benefit from measuring moral cognition in larger MS cohorts with different disease phenotypes and applying comprehensive neuropsychological batteries to understand the relationship between moral cognition and other cognitive and socio-emotional domains, as well as clinical, cultural, and demographic characteristics.

Regarding the assessment of moral judgment, different assessment tools have been employed across the studies and may yield different moral judgments [32]. Standardizing tasks would be of help, since different tasks (e.g., task type and complexity) render it difficult to compare results across the studies. Moreover, adopting an ecological approach and increasing the apparent validity of dilemmas might be possible by adapting immersive virtual environments that more resemble real-life settings compared to stories that might not reflect how individuals behave in a more enriched social context [37,111].

**Figure A1:**
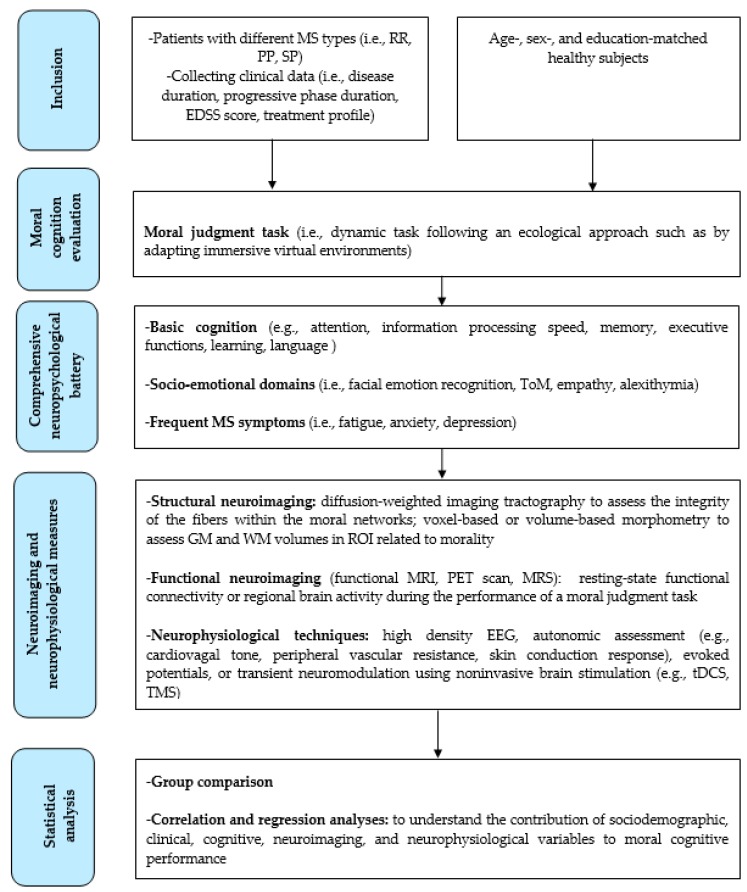
A proposed roadmap for future research addressing moral cognition in multiple sclerosis. EDSS: Expanded Disability Status Scale; EEG: electroencephalography; MRI: magnetic resonance imaging; MRS: magnetic resonance spectroscopy; MS: multiple sclerosis; RR: relapsing remitting; PET: positron emission tomography; PP: primary progressive; ROI: region of interest; SP: secondary progressive; tDCS: transcranial direct current stimulation; TMS: transcranial magnetic stimulation; ToM: theory of mind.

Furthermore, in the absence of any study assessing the underlying mechanisms of moral judgment in this population, future application of different neuroimaging modalities, such as functional magnetic resonance imaging, diffusion-weighted imaging tractography, or voxel-based morphometry, would be of great interest in order to decipher the neural underpinnings of morality in MS patients. Neurophysiological techniques, such as high-density electroencephalography [112], autonomic assessment (e.g., cardiovagal tone, peripheral vascular resistance, skin conduction response) [34,35,36,37,38], or transient neuromodulation using noninvasive brain stimulation (NIBS), may also serve this purpose [113]. A research design for future studies is suggested in Figure A1.

Finally, if future research concludes a serious impact of aberrant moral judgment on patients’ daily life and social function, attention should be paid on finding therapeutic solutions. Studies in healthy volunteers and other clinical populations have tested the effects of (i) pharmacological molecules, (ii) psychotherapies, and (iii) NIBS techniques on moral judgment. For instance, the administration of hormones (i.e., testosterone) [114,115], neuropeptides (e.g., oxytocin) [43], or other drugs that enhance neurotransmission (i.e., serotonin-specific reuptake inhibitors [43,44], dopamine precursors [44], noradrenergic beta-adrenoceptor antagonists [46], gamma aminobutyric acid (GABA) agonists [116]) were found to modulate the pattern of moral judgment, but the direction of moral judgment changes (utilitarian versus deontological) varied across the studies. Other than medications, NIBS techniques have recently emerged as appealing therapeutic interventions in several neuropsychiatric conditions, including MS [7,113,117,118,119,120], and might have their place in modulating moral judgment deficits. By transcranially applying an electric current or a magnetic field, NIBS modulates the functions of several cerebral areas [7,113,117,118,119,120]. The application of NIBS over cortical areas that take part in moral judgment networks, such as dorsolateral PFC [121,122,123,124,125]), ventral PFC [126], or TPJ [127,128,129,130,131], have also resulted in some changes in moral judgment performance. However, the protocols differed in their design, stimulation parameters, and targeted areas, and yielded conflicting and sometimes negative results. Although incongruent, these preliminary findings give some hope for the possibility of using these techniques to improve moral judgment. The implication of physiological arousal in moral judgment suggests a third category of interventions. In fact, mindfulness-based interventions, biofeedback, and cognitive behavioral therapy or some of its components (i.e., cognitive reappraisal techniques) have been applied in MS [132,133,134] and might be helpful to train bodily signals, which seem to play a role in moral reasoning. However, facing all these possible interventions, one should keep in mind that shaping morality could raise serious ethical concerns [135], and setting regulations is warranted to meet this concern [18].

## Figures and Tables

**Table 1:** A summary of studies assessing moral cognition in patients with multiple sclerosis (MS).

Study	Gleichgerrcht et al., 2015 [61]	Patil et al., 2017 [62]	Realmuto et al., 2018 [63]
Patients’ demographic and clinical data	38 RR MS patients	38 consecutive RR MS patients	45 RR MS patients
	87.30% females	86.80% females	68.89% females
	Mean age: 42.3 ± 11.3 years	Mean age: 42.3 ± 11.3 years	Mean age: 34.22 ± 7.65 years
	All receiving immunomodulatory drugs	All receiving immunomodulatory drugs	Immunomodulatory treatment: details N/A
	Mean education level: 15.4 ± 2.8 years	Mean education level: 15.4 ± 2.8 years	Mean education level: 13.49 ± 2.46 years
	Mean EDSS score [94]: 1.66 ± 1.6	Mean EDSS score [94]: 1.66 ± 1.6	Mean EDSS score [94]: 2.06 ± 1.46
	Mean disease duration: 1.6 ± 8.7 years	Mean disease duration: 10.60 ± 8.7 years	Mean disease duration: 9.72 ± 6.22 years
	Mean number of relapses: 3.4 ± 1.92	Mean number of relapses: 3.4 ± 1.92	Mean number of relapses: details N/A
	Mean MSSS score [95]: 2.35 ± 2.4	Mean MSSS score [95]: 2.35 ± 2.4	Mean MSSS score [95]: 2.85 ± 2.59
Healthy control group	38 age-, gender-, and education-matched healthy controls	38 age-, gender-, and education-matched healthy controls	45 age-, gender-, and education-matched healthy controls
Assessment tool for moral judgement	Moral dilemma task: a series of eight vignettes from Greene et al.’s battery [21,27] presenting situations measuring moral permissibility, emotional reactivity, and moral relativity	Moral intent task: 24 unique stories adapted from Young et al. 2010 [74]	Moral dilemmas including instrumental and incidental conditions [57]
Other measures	Alexithymia: TAS [96] Empathy: IRI [97]	Alexithymia: TAS [96] Empathy: IRI [97]	Non-social cognition evaluation: BICAMS [98], Cognitive Estimation task [99], and Stroop test [100] Social cognition evaluation: Ekman-60 Faces test, RMET, and Story-based Empathy task [101,102]. Quality of life: MuSIQoL [103] Fatigue: FSS [104] Depression and anxiety: HADS [105]
Group comparison	Patients exhibited reduced moral permissibility, increased moral relativity, increased emotional reactivity, low empathy, and high alexithymia rating compared to healthy controls	Compared to healthy controls, patients had comparable levels of moral judgement but exhibited reduced moral permissibility, increased moral relativity, increased emotional reactivity, low empathy and high alexithymia ratings	No significant group differences in the levels of moral judgment (rate of yes/no response in dilemmas resolution; attribution of emotional valence to moral actions) but had lower moral permissibility and emotional arousal (for the instrumental dilemmas 13.33% of patients had poor moral judgement performance) 77.6% of patients had non-social cognitive deficits (i.e., executive domains) 24% of patients had social cognitive deficits
Correlation analysis	Significant positive correlation between moral reactivity and MSSS scores Significant positive correlation between moral permissibility, empathy, and alexithymia scores	No significant correlation between moral judgement and empathy or alexithymia measures Tendency toward negative correlation between appropriateness of intentional harm and alexithymia (did not survive statistical corrections) Significant negative correlation between appropriateness of intentional harm and empathy measures, perspective taking, and empathic concern (did not survive statistical corrections)	Significant correlations between the attribution of emotional valence and mentalizing (did not survive statistical corrections) No other correlations between moral judgment and clinical, basic cognition, or social cognition measures

BICAMS: Brief International Cognitive Assessment for Multiple Sclerosis battery; EDSS: Expanded Disability Status Scale; FSS: Fatigue Severity Scale; HADS: Hospital Anxiety and Depression Scale; IRI: Interpersonal Reactivity Index; MS: Multiple sclerosis; MuSIQoL: Multiple Sclerosis International Quality of Life; MSSS: Multiple Sclerosis Severity Score; N/A: Not available; RMET: Reading the Mind in the Eyes Test; RR: Relapsing remitting; TAS: Toronto Alexithymia Scale.
